# Benign Phyllodes Tumors: Comparison of Prognosis among Three Different Surgical Approaches

**DOI:** 10.1155/2023/1682084

**Published:** 2023-09-20

**Authors:** Ying Jiang, Bo Wang, Jun Kang Li, Shi Yu Li, Rui Lan Niu, Nai Qin Fu, Jiao Jiao Zheng, Gang Liu, Zhi Li Wang

**Affiliations:** ^1^School of Medicine, Nankai University, 94 Weijin Road, Tianjin 300071, China; ^2^Department of Ultrasound, Chinese People's Liberation Army General Hospital, 28 Fuxing Road, Beijing 100853, China; ^3^Department of Radiology, Chinese People's Liberation Army General Hospital, 28 Fuxing Road, Beijing 100853, China

## Abstract

**Purpose:**

To evaluate the prognosis of patients with benign phyllodes tumors (PTs) treated by different surgical methods and to explore the influencing factors of local recurrence.

**Methods:**

We retrospectively analyzed 215 benign PTs from 193 patients who underwent surgery at Chinese PLA General Hospital between October 2008 and December 2020. We stratified our analysis according to surgical factors and explored the clinicopathological factors to influence local recurrence.

**Results:**

Among 193 patients, a total of 17 (8.8%, 17/193) recurred during follow-up. There were 89 patients in the US-VAE group, of whom 6 (6.7%) recurred; 8 of 57 patients (14%) in the local lumpectomy group recurred, while 3 of 47 patients (6.4%) in the extended lumpectomy group recurred (*P*=0.252). Multivariate logistic regression analysis showed that tumor diameter, mitosis, and history of breast myoma were independent risk factors for tumor recurrence (*P*=0.005, *P*=0.006, and *P*=0.004, respectively). The intraoperative blood loss, operation time, and scar length of the US-VAE group were shorter than those of the other two groups (*P* < 0.05).

**Conclusion:**

Negative surgical margins of benign PTs can obtain similar prognosis as negative surgical margins >10 mm. Therefore, we recommend that a follow-up observation policy be adopted for patients with unexpected benign PTs, rather than unnecessary open surgical resection. Patients' maximum tumor diameter, mitosis, and fibroadenoma history were independent predictors for recurrence of benign PTs.

## 1. Introduction

Benign phyllodes tumors (PTs) of the breast are rare fibroepithelial tumors, accounting for less than 1% of all primary breast tumors [1]. The World Health Organization (WHO) classifies PTs into benign, borderline, and malignant according to the pathological characteristics of tumors. The proportions of the three subtypes are 60%–75%, 13%–26%, and 10%–20%, respectively [2]. For women, it mainly occurs between the ages of 35 and 55 years [3]. Surgery is the main treatment for this disease. The surgical methods include ultrasound-guided vacuum-assisted resection (US-VAE), local mass resection, and extended mass resection. Both previous findings and the national comprehensive cancer network (NCCN) recommend that the negative surgical margin of PTs must be no less than 10 mm regardless of the surgical approach [4].

The low incidence of PTs and the lack of large-scale clinical data lead to our current limited understanding of it. The recurrence rate of benign PTs is 0–20.8%, which is lower than that of borderline or malignant PTs [5, 6]. Since benign PTs have a better prognosis, can patients be spared negative margin surgery larger than 10 mm? In view of this, this study provides the first systematic comparison of the three surgical modalities for benign phyllodes tumors. Can emerging minimally invasive US-VAE techniques be comparable to traditional open surgery? What are the risk factors that affect the local recurrence of benign tumors? This research will answer the abovementioned questions separately.

## 2. Methods

### 2.1. Patient Enrollment

This retrospective study was approved by the Medical Ethics Committee of PLA General Hospital and waived the requirement for informed consent. We evaluated the pathological data and clinical outcomes of patients who underwent breast mass surgery in our hospital from October 2008 to December 2020, including age, menopause, history of fibroadenoma, tumor size, ultrasound findings, histological characteristics, tumor recurrence, and operation type. The inclusion criteria for the enrolled patients were as follows: (a) patients underwent one of the following three surgeries: US-VAE, local mass resection, or extended mass resection. (b) The pathology of breast mass resection was benign PTs. Exclusion criteria were as follows: (a) including carcinoma in situ and invasive breast cancer and (b) previous breast cancer history and unavailable follow-up data. This study stratified patients according to surgical factors and analyzed the clinicopathological risk factors of recurrence in patients with benign PTs.

### 2.2. Ultrasound-Guided Vacuum-Assisted Excision Procedure

The US-VAE procedures were mainly performed by a well-organized team headed by Zhi Li Wang, using the iU22 ultrasound system (Philips Medical Systems, Andover, Massachusetts), with an L12-5 linear array probe, to determine the position and boundary of the lesions. The optimal insertion site and the needle track are designed based on ultrasound examination. Local infiltration anesthesia around the tumor was performed. Then, we used the No. 7 EnCor Probe® system (EnCor® inserts MR, SenoRx, Allso Viejo, CA) directly into the tumor or behind the tumor through a 2-3 mm incision, cut, and automatically transported the tissue sample to the collection basket. When the ultrasound examination confirms that there is no obvious lesion image, we stop cutting. Then, we take out the rotating cutting needle and apply partial pressure to prevent bleeding. We then immediately performed an ultrasound scan after surgery to ensure there are no residual lesions or hematomas. An adhesive elastic band was used for 24–48 h. The treatment time depends on the size of the lesion and is defined as the period from the first incision to the end of the last incision.

### 2.3. Local Lumpectomy Procedure

We determine the location and size of the tumor by palpation. After the local anesthesia is completed, the skin and subcutaneous tissue are incised. The tumor is completely removed by carefully separating the surrounding area of the tumor along the breast tissue. Before suturing the skin, we thoroughly rinse the surgical area and stop bleeding and store instruments and dressings. Finally, an elastic bandage was used to compress the postoperative incision.

### 2.4. Extended Lumpectomy Procedure

After palpating to determine the location and size of the tumor, local anesthesia (or general anesthesia) is performed. The skin and subcutaneous tissue were incised on the surface of the breast tumor, carefully separated from the pectoralis major fascia along 10 mm around the tumor, and the tumor was completely removed. Before suturing the skin, we flushed the surgical field, stopped bleeding thoroughly, and counted the instruments and dressings without error. Then, we used an elastic bandage to compress the postoperative incision.

### 2.5. Follow-up

The follow-up was censored in December 2020, and the follow-up of all patients was performed by reviewing the electronic system, text messages, or telephone calls (one of the methods or more) of the outpatient clinic of our hospital. Patients' follow-up time and follow-up results (physical examination + ultrasound or MRI examination) were recorded.

### 2.6. Data and Statistical Analysis

The age used for each patient was the age at the time of diagnosis. Lesion size on US was defined by the maximum diameter. The follow-up period was defined as the time from the confirmation of benign PTs by surgery to the last clinical follow-up. Recurrence was defined as clinical and sonographic tumor recurrence within the previously resected area.

All statistical analyses were performed using SPSS 23.0, standard version (SPSS Inc., Chicago, IL). Statistical comparisons were performed using the *χ*^2^-test or Fisher's exact test for categorical variables and the Kruskal–Wallis test for continuous variables. The relationship between each clinical factor and local recurrence was screened by univariate analysis. The logistic multiple regression analysis was subsequently used to calculate and analyze the independent influencing factors of local recurrence. *P* values <0.05 were considered statistically significant.

## 3. Results

### 3.1. Clinicopathologic Features

**Figure 1:**
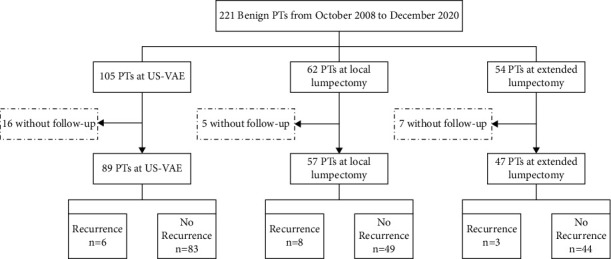
Flowchart illustrating the selection process of the study population.

**Figure 2:**
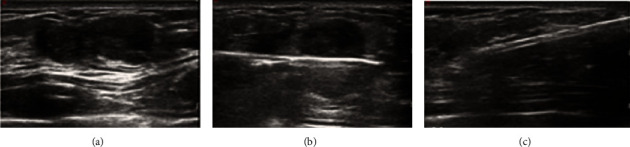
A lesion in a 36 years woman, which was demonstrated to be PTs by pathology. (a) Ultrasound imaging showed a hypoechoic mass; (b) the probe in the mass; (c) complete resection of the mass.

A total of 243 benign PTs in 221 female patients were screened out. Among these cases, 28 without follow-up were excluded. Therefore, a total of 215 benign PTs (Figure 1) from 193 patients were finally included, 89 of which underwent US-VAE (Figure 2), 57 underwent local lumpectomy, and 47 underwent extended lumpectomy. The demographic characteristics, including age, history of breast fibroids, image examination (including sonographic findings), and surgical records, were extracted from the original resumes. The clinicopathological characteristics are listed in Table 1.

### 3.2. Recurrence Rate

Among the 193 patients, 17 cases (8.8%, 17/193) recurred during the follow-up period; in the US-VAE group, 6 out of 89 patients (6.7%) relapsed; in the local lumpectomy group, 8 out of 57 patients (14%) recurred, while 3 out of 47 tumors in the extended lumpectomy group (6.4%) recurred (*P*=0.252). Recurrent cases are summarized in Table 2. All recurrent lesions were treated surgically and pathologically diagnosed. One of the recurrences was a malignant phyllodes tumor, and 16 cases were benign PTs. Among these 17 relapsed patients, no distant metastasis occurred during the subsequent follow-up period.

Univariate analysis showed that tumor diameter, mitosis, and history of breast fibroma may be potential risk factors for recurrence (Table 3; *P* < 0.05). Age, menopause, lump location, echo pattern, lesion edge, blood flow, BI-RADS categories, and surgical methods were not significantly different between the groups with and without recurrence (*P*=0.350, 0.218, 0.163, 0.177, 0.409, 0.365, 0.861, and 0.252, respectively). Multivariate logistic regression analysis revealed that the diameter of the lump, mitoses, and history of breast fibroids are independent risk factors for tumor recurrence. The results are presented in Table 4.

### 3.3. Complications

Complications include hematoma, wound oozing, infection, and ecchymosis. A total of 8 patients in the US-VAE group developed complications, of which 6 developed hematomas and 2 developed skin ecchymosis. A total of 6 patients in the local lumpectomy group had complications, of which 1 had hematoma, 2 had wound exudative infection, and 3 had skin ecchymosis. Seven patients in the extended lumpectomy group had complications, 2 patients had ecchymosis and hematoma, and 3 patients had wound exudative infection. (*P*=0.572, Table 1) The intraoperative blood loss, operation time, and wound length in the group US-VAE were shorter than those in the other two groups (*P* < 0.05), as detailed in Table 5.

## 4. Discussion

The study aimed to systematically compare the effects of three surgical methods on the recurrence of benign PTs. NCCN guidelines recommend a negative surgical margin distance of no less than 10 mm for all pathologic types of PTs. However, few datasets support this practice. In our study, after a median follow-up of 39 months, 17 patients had local recurrence. Among them, there were 16 benign PTs and 1 malignant PT. The difference in recurrence of benign PTs between different surgical approaches was not statistically significant, which illustrated that a negative resection margin greater than 10 mm could not reduce the recurrence rate of benign PTs. An analysis [7] of 1,702 patients enrolled in 12 studies also showed that the recurrence rate of benign PTs after surgery was very low and that 112 cases of 1,052 benign phyllodes tumors recurred. When the margin is positive, the recurrence rate increases. Graña López et al. [8], by analyzing 160 benign breast nodules (11 lesions corresponded to benign PTs) resected from 2007 to 2016, concluded that resection of benign PTs by vacuum-assisted percutaneous excision did not increase the risk of recurrence compared with surgery. It should be noted that the 89 patients who underwent US-VAE included in this study were not diagnosed with phyllodes tumors preoperatively, so these lesions were benign PTs accidentally diagnosed by US-VAE. The second International Consensus Conference on lesions of uncertain malignant potential in the breast (B3 lesions) also recommended vacuum-assisted biopsy system treatment for the lesion which lacks high-risk cytological features, although more prolonged observation is necessary [9, 10]. Therefore, although our sample size was small, these results suggest that surgical margin status does not appear to be predictive of local recurrence and that increasing the negative distance to the surgical margin does not confer greater benefit to patients with breast PTs. At the same time, we recommend a follow-up observation policy for patients with unexpected benign PTS, rather than unnecessary open surgical resection.

PTs of the breast may lead to the degradation or upgradation of pathological types in the process of recurrence and metastasis. In our study, it was observed that after local recurrence, the pathological type of one patient was upgraded from benign to malignant. This is similar to Tan's previous report. In his study of 353 cases of breast phyllodes, he found that the pathology of 14 cases of breast phyllodes upgraded from benign to marginal after recurrence [11]. Therefore, in clinical practice, we should try to avoid the recurrence of patients to avoid pathological upgrading. At present, the causes of pathological changes after recurrence or metastasis of breast PTs are not clear, and its mechanism and pathways need to be further studied. There may be new genetic changes in recurrent phyllodes. In a previous study, only 3 of the 9 benign phyllodes showed histological upgrading at the time of recurrence, but 6 (67%) of the 9 histological benign tumors obtained new gene changes related to borderline/malignant phenotypes (+1q, +7p, −9p, and −13) [12].

Different authors found a broad variety of predictors, although with varying statistical power. In the present study, multivariate logistic regression analysis revealed that the diameter of the lump, mitoses, and history of breast fibroids are independent risk factors for tumor recurrence. First, although the prediction of local recurrence rates of PTs based on tumor size remains controversial, in recent studies, smaller PTs still show lower recurrence rates than larger PTs [13, 14]. The results of our study suggest that the median tumor size of the US-VAE group was 2.27 cm, which is significantly smaller than that of the other two groups. The reason for the difference in sizes is when the benign breast tumor is small, patients are more inclined to choose minimally invasive surgery. US-VAE has a high complete resection rate and can leave smaller scars [15]. When the tumor diameter is relatively large, US-VAE is less suitable as a treatment modality. It may be because those with relatively large tumor volume require multiple excisions, which may easily cause gas accumulation in the tumor cavity, affect B-ultrasound observation, and increase the risk of tumor recurrence [16]. Therefore, US-VAE is more suitable for benign PTs with a maximum diameter of <3 cm. Others have a similar opinion about the tumor size cutoff when treating benign PTs with US-VAE, while some scholars also believe that the cutoff value of tumor size using the US-VAE method should be 3.3 cm [17, 18]. Patients with tumor size ≥3 cm and <3 cm were evaluated separately, and there was a statistically significant difference in the local recurrence rate, indicating that patients with breast tumors ≥3 cm are prone to recurrence. Therefore, for benign PTs with larger diameters, extended lumpectomy seems to be a more effective method for reducing the local recurrence rate than US-VAE and local lumpectomy.

To date, the number of mitotic figures also determines the prognosis of PTs to some extent. In this study, patients with high mitosis figures had an OR value of 10.881 compared with those with low mitoses' figures. This shows that for benign PTs, as the number of mitotic images increases, the possibility of recurrence is greater. At the same time, we found that benign PTs with a history of fibroadenoma had a higher recurrence rate than those without a history of fibroadenoma. Therefore, when patients have a history of fibroadenoma, a regular review should be adhered to guard against secondary or even multiple recurrences. Given that fibroadenoma and PTs belong to the same category as fibroepithelial breast tumors and have similar clinical symptoms, imaging manifestations, and histomorphology. Relevant studies have suggested that they may be partially correlated [19]. In a recent study, exome sequencing of 22 PTs and targeted sequencing of 100 fibroepithelial neoplasms revealed the genetic profile of fibroepithelial neoplasms, with MED12 (73%) and RARA (32%) mutations common in fibroadenoma patients and in all pathologically classified PTs' patients [20]. Notably, mutations in FLNA (28%), SETD2 (21%), and KMT2 (9%) were observed only in PTs, suggesting a role in driving PTs' development. Therefore, we speculated that some patients with a history of fibroadenoma had stromal cell mutation during recurrence, and fibroadenoma was upgraded to PTs due to tumor disease. This study also shows that US-VAE treatment of PTs is superior to the conventional open surgery in terms of wound length, blood loss, operation time, postoperative scar size, and breast beauty. There was no statistically significant difference in the incidence of postoperative complications among the three surgical methods. These conclusions have also been confirmed in previous studies [21, 22].

This study has some limitations. First, this is a single-center retrospective study, and the relatively small number of patients recruited will lead to inevitable inherent bias. For example, patients treated with US-VAE are more likely to have smaller tumors. Although the data were included continuously, we also considered these factors in the multivariate analysis, and the study still included other factors that could not be adjusted. Second, in the present study, this exclusion may have led to biased recurrence rate estimates when loss to follow-up did not occur at random or was associated with a hazard of death. Finally, a further study with a larger sample size and longer follow-up is necessary.

## 5. Conclusions

Negative surgical margins of benign PTs can obtain similar prognosis as negative surgical margins >10 mm. Therefore, we recommend that a follow-up observation policy be adopted for patients with unexpected benign PTs, rather than unnecessary open surgical resection. Patients' maximum tumor diameter, mitosis, and fibroadenoma history were independent predictors for recurrence of benign PTs.

## Figures and Tables

**Table 1:** Clinicopathological characteristics of patients.

Characteristics	US-VAE group (*n* = 89)	Local lumpectomy group (*n* = 57)	Extend lumpectomy group (*n* = 47)	*P* value
Age (y)	32.764 ± 9.807	39.825 ± 14.069	40.894 ± 12.936	<0.001
Menopause	8 (9)	10 (17.5)	10 (21.3)	0.114
Lump location				0.877
Left	35 (39.3)	26 (45.6)	21 (44.7)	
Right	42 (47.2)	25 (43.9)	22 (46.8)	
Both side	12 (13.5)	6 (10.5)	4 (8.5)	
Maximum diameter				<0.001
≥3 cm	14 (15.7)	35 (61.4)	28 (59.6)	
<3 cm	75 (84.3)	22 (38.6)	19 (40.4)	
Mean ± SD	2.27 ± 0.75	3.45 ± 1.71	3.60 ± 2.03	
Hypoechoic pattern	86 (96.6)	52 (91.2)	38 (80.9)	0.009
Clear lesion edge	79 (88.8)	46 (80.7)	42 (89.4)	0.306
Blood flow				0.061
0-I level	56 (62.9)	43 (75.4)	38 (80.9)	
II-III level	33 (37.1)	14 (24.6)	9 (19.1)	
BI-RADS category				0.120
3	55 (61.8)	35 (61.4)	23 (48.9)	
4	34 (38.2)	21 (36.8)	21 (44.7)	
5	0 (0)	1 (1.8)	3 (6.4)	
Mitoses/10HPF				0.093
0–4	84 (94.4)	52 (91.2)	39 (83.0)	
5–9	5 (5.6)	5 (8.8)	8 (17.0)	
History of fibroids	31 (34.8)	18 (31.6)	22 (46.8)	0.242
Complications	8 (9)	6 (10.5)	7 (14.9)	0.572
Follow-up period (mo)	39 (6–123)			

US-VAE: ultrasound-guided vacuum-assisted excision.

**Table 2:** Patient and tumor characteristics of the 17 recurred cases.

Case number	Surgical approach	Age (y)	Maximum diameter (cm)	Time to postoperative recurrence (mo)	Recurrent tumor pathology	Tumor size after recurrence (cm)
1	US-VAE	49	3.2	12	Benign PT	1.7
2	US-VAE	23	3.6	10	Benign PT	2.3
3	US-VAE	22	4.9	6	Benign PT	3.5
4	US-VAE	22	2.7	8	Benign PT	1.2
5	US-VAE	36	2.3	9	Benign PT	1.8
6	US-VAE	55	2.5	96	Benign PT	3.1
7	Local lumpectomy	42	3	24	Benign PT	5.4
8	Local lumpectomy	41	8	10	Benign PT	1.2
9	Local lumpectomy	35	4.2	6	Benign PT	3.2
10	Local lumpectomy	48	5.4	10	Malignant PT	1.3
11	Local lumpectomy	37	3.1	2	Benign PT	1.5
12	Local lumpectomy	22	6.8	2	Benign PT	1.6
13	Local lumpectomy	46	4	60	Benign PT	2.1
14	Local lumpectomy	64	2.3	12	Benign PT	1.5
15	Extend lumpectomy	41	4.7	12	Benign PT	1.8
16	Extend lumpectomy	12	8.4	6	Benign PT	5.9
17	Extend lumpectomy	43	3.2	8	Benign PT	1.3

US-VAE: ultrasound-guided vacuum-assisted excision; PT: phyllodes tumor.

**Table 3:** Univariate analysis of influencing factors of postoperative local recurrence.

Characteristics	No recurrence (*n* = 176)	Recurrence (*n* = 17)	*P* value
Age (y)	36.76 ± 12.423	37.53 ± 13.666	0.845
Menopause	24 (13.6)	4 (23.5)	0.218
Lump location			0.163
Left	74 (42.0)	8 (47.1)	
Right	84 (47.7)	5 (29.4)	
Both side	18 (10.2)	4 (23.5)	
Maximum diameter			0.002
≥3 cm	64 (36.4)	13 (76.5)	
<3 cm	112 (63.6)	4 (23.5)	
Hypoechoic pattern	162 (92.0)	14 (82.4)	0.177
Clear lesion edge	153 (86.9)	14 (82.4)	0.409
Blood flow			0.365
0-I level	126 (71.6)	11 (64.7)	
II-III level	50 (28.4)	6 (35.3)	
BI-RADS category			0.861
3	102 (58.0)	11 (64.7)	
4	70 (39.8)	6 (35.3)	
5	4 (2.2)	0 (0)	
Mitoses/10HPF			<0.001
0–4	167 (94.9)	8 (47.1)	
5–9	9 (5.1)	9 (52.9)	
History of fibroids	59 (33.5)	12 (70.6)	0.003
Surgical methods			0.252
US-VAE	83 (47.2)	6 (35.3)	
Local lumpectomy	49 (27.8)	8 (47.1)	
Extend lumpectomy	44 (25.0)	3 (17.6)	

US-VAE: ultrasound-guided vacuum-assisted excision.

**Table 4:** Multivariate logistic regression analysis of influencing factors of postoperative local recurrence.

Variables	*β*	SE	Wald *χ*^2^	*P*	OR	OR 95% CI
Age ≥40	0.257	0.611	0.176	0.674	1.292	0.391–4.277
Maximum diameter ≥3 cm	2.024	0.717	7.967	0.005	7.572	1.857–30.884
Mitoses/10HPF	2.387	0.860	7.702	0.006	10.881	2.016–58.724
History of fibroids	1.804	0.625	8.348	0.004	6.077	1.787–20.667
Surgical methods			5.369	0.068		
Local lumpectomy/US-VAE	0.013	0.715	0.000	0.985	1.014	0.250–4.112
Extend lumpectomy/US-VAE	−2.150	1.035	4.318	0.038	0.116	0.015–0.885

US-VAE: ultrasound-guided vacuum-assisted excision.

**Table 5:** Comparison of operation-related indexes between the three groups.

	*N*	Intraoperative blood loss (ml)	Operative time (min)	Wound length (mm)
US-VAE group	89	7.97 ± 3.19	22.45 ± 8.55	4.48 ± 0.89
Local lumpectomy group	57	13.68 ± 7.17	38.47 ± 10.36	32.98 ± 13.75
Extend lumpectomy group	47	25.43 ± 12.85	57.34 ± 19.08	38.13 ± 18.47
*χ* ^2^		79.00	114.51	151.15
*P*		0.000	0.000	0.000

US-VAE: ultrasound-guided vacuum-assisted excision.

## Data Availability

The data used to support the findings of this study are available from the corresponding author upon request.
